# Complications of central venous catheter in patients transplanted with hematopoietic stem cells in a specialized service

**DOI:** 10.1590/1518-8345.0547.2698

**Published:** 2016-06-07

**Authors:** Lidiane Miotto Barretta, Lúcia Marinilza Beccaria, Cláudia Bernardi Cesarino, Maria Helena Pinto

**Affiliations:** 2 RN, Centro de Educação Permanente, FUNFARME, São José do Rio Preto, SP, Brazil.; 3 Adjunct Professor, Departamento de Enfermagem Geral, Faculdade de Medicina de São José do Rio Preto, São José do Rio Preto, SP, Brazil.; 4 Professor, Departamento de Enfermagem Geral, Faculdade de Medicina de São José do Rio Preto, São José do Rio Preto, SP, Brazil.

**Keywords:** Central Venous Catheters/Adverse Effects, Hematopoietic Stem Cell Transplantation, Bone Marrow Transplantation

## Abstract

**Objective:**

to identify the model, average length of stay on site and complications of central venous catheter in patients undergoing transplant of hematopoietic stem cells and verify the corresponding relationship between the variables: age, gender, medical diagnosis, type of transplant, implanted catheter and insertion site.

**Method:**

a retrospective and quantitative study with a sample of 188 patients transplanted records between 2007 and 2011.

**Results:**

the majority of patients used Hickman catheter with an average length of stay on site of 47.6 days. The complication fever/bacteremia was significant in young males with non-Hodgkin's lymphoma undergoing autologous transplant, which remained with the device for a long period in the subclavian vein.

**Conclusion:**

nurses should plan with their team the minimum waiting time, recommended between the catheter insertion and start of the conditioning regimen, as well as not to extend the length of time that catheter should be on site and undertake their continuing education, focusing on the prevention of complications.

## Introduction

Hematopoietic Stem Cell Transplantation (HSCT) is a therapy used for treatment of two conditions, one comprising non-malignant diseases resulting from bone marrow function failure or cells derived from bone marrow, in which transplant is used to replace a defective tissue, and in another condition, most prevalent, used for neoplastic diseases, particularly malignant^1^ ^-^ ^2^. The term HSCT is used to replace Bone Marrow Transplantation (BMT), since it describes the current form of the procedure, which involves Hematopoietic Stem Cells (HSC) directly aspirated from bone marrow, peripheral stem cells mobilized from the marrow compartment into the peripheral blood, or from the umbilical cord blood^3^ ^-^ ^4^.

There are two main types of transplant: autologous, when the patient is his own donor and allogeneic, when someone is compatible as a donor, which may be a family member, a volunteer, or cells from stored umbilical cord blood. Rarely, patients may have an identical twin, allowing the syngeneic transplant. For all types of HSCT, a tunneled Central Venous Catheter (CVC) is usually placed for the administration of chemotherapy, stem cell infusion, intravenous medication, electrolyte supplements, nutritional support and blood products^1^.

Some procedures are essential after insertion of Central Venous Catheter, because, if there is loss of permeability of the pathways and it is not treated quickly, there will be a permanent loss of access. In addition, there will also be increased morbidity resulting from new catheterizations, thereby raising the risk of infection due to the formation of fibrin and adherence of bacteria and fungi, worsened by the large number of catheter manipulations, amount of lumens, type of clothing and age of the patient^5^.

The granulocytopenia secondary to the conditioning chemotherapy determines the risk of catheter-related infections, which may serve as an entry into the blood circulation, leading to bacteremia, fungemia, and consequently to septic shock and death. The risks of infection and the spectrum of infectious syndromes differ according to the type of transplant, conditioning regimen, type of implant of stem cells and therapies used after the procedure^1^ ^,^ ^6^.

In HSCT units, there are situations that culminate in the early removal of the catheter, resulting in rework that, in addition to increasing the chance of complications, directly reflects on the cost and course of treatment, and still causes great distress to patients and their families and/or caregivers. Therefore, it is important to research the causes of removal of central venous access in patients during HSCT process, as well as which factors influence these causes, so that staff can develop a safer care.

Given the above, this study aimed to identify the model, average length of stay on site and complications of CVC in patients undergoing HSCT in a specialized service, and verify the corresponding relationship between the variables: age, gender, medical diagnosis, transplant performed, implanted catheter and insertion site.

## Method

Exploratory and retrospective study with a quantitative approach, performed in the Bone Marrow Transplantation Unit of a general and teaching hospital in a municipality in the State of São Paulo, with a sample of 188 patients, out of 221 patients transplanted, selected by means of medical records and 249 CVC control files, from January 2007 to December 2011.

Inclusion criteria were: medical records of patients undergoing HSCT and catheter control files that were fully completed, used by the nursing service, and containing patient identification, medical diagnosis, type of transplant, catheter model, date of implant, history of manipulations, as well as the date, reason and professional responsible for removal of the catheter. To characterize the subjects, it was used the following variables: gender, age, medical diagnosis, type of transplant, model, insertion site and length of stay with the catheter.

Correspondence analysis was the statistical test used, a multivariate technique in which similar categories are close to each other and show associations that would not be detected by means of comparison between nominal variables^7^.

## Results

Of the 188 patients, 58% (110) were male. The identified complications were fever/ bacteremia 13.65% (34), accidental CVC removal 7.63% (19), peri-insertion tunelitis/hyperemia 6.42% (16), subcutaneous leakage 6.02% (15), infection 5.22% (13), exteriorization of catheter fixation cuff 5.22% (13), catheter obstruction 4.81% (12), arrhythmia 0.40% (1). Had no complications during the period of time that the catheter was on site 42.55% (106) and 8.03% (20) died in the course of treatment due to causes unrelated to the implanted catheter.

A higher prevalence of staying with the catheter until hospital discharge was observed among females, which showed correspondence association with tunelitis/hyperemia, followed by loss of access due to accidental removal. Fever/bacteremia, obstruction, exteriorization of catheter fixation cuff (Hickman) and subcutaneous extravasation were more significant among males, with a greater number of deaths. The association between infection and gender was not statistically significant.

Hickman catheter was the most commonly used, with 70% (175), followed by Double Lumen (DL) catheter 7x20cm with 16% (40), hemodialysis catheter (SHILLEY^R)^ with 11% (27) and others with low incidence of implantation, 3% (7). Hickman catheter showed a higher association with obstruction, infection, tunelitis/hyperemia, in addition to the exteriorization of catheter fixation cuff, which is its characteristic. DL catheter was associated with extravasation and the most commonly used in patients who died after transplant, whereas hemodialysis catheter was associated with fever/bacteremia and Permicath^R^ catheter, 0.01% (3), did not present complications.

In allogeneic HSCT, 21.80% (41), there was a higher incidence of subcutaneous extravasation and tunelitis/hyperemia, whereas in autologous, 78.19% (147), it was observed a higher incidence of fever/bacteremia, accidental removal of the catheter, infection, exteriorization of catheter fixation cuff and arrhythmia. The type of transplant was not statistically significant in relation to obstruction of the catheter.

**Figure 1:**
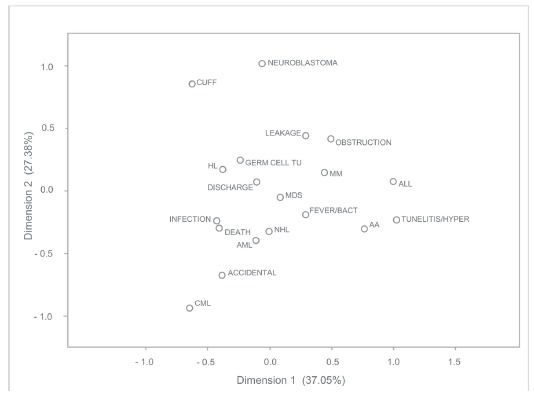
Correspondence between complications of CVC and medical diagnostics of HL NHL, CEL GERM TU, Acute Lymphoid Leukemia (ALL), Chronic Myeloid Leukemia (CML), AML, AA and Myelodysplastic Syndromes (MDS). São José do Rio Preto, SP, Brazil, 2007-2011

With regard to medical diagnostics, Acute Myeloid Leukemia (AML) and non-Hodgkin's lymphoma (NHL) have been mainly associated with infection and death. Patients with Aplastic Anemia (AA) presented complications related to the insertion site, such as tunelitis/hyperemia. Diagnosis of multiple myeloma (MM) was associated with complications related to catheter obstruction and subcutaneous leakage. On the other hand, patients who were discharged from hospital without complications related to CVC, had Hodgkin's Lymphoma (HL) and Germ Cell Tumor (GERM CEL TU) as underlying diseases, as shown in Figure 1.

Regarding age, children (under 10 years) presented a higher association with fever/infection and bacteremia. Patients aged 11-20 years exhibited a tendency to accidental removal of the device. Tunelitis/hyperemia occurred mainly at the age between 31 and 40 years and this was the group of patients who achieved most successful transplant.

**Figure 2:**
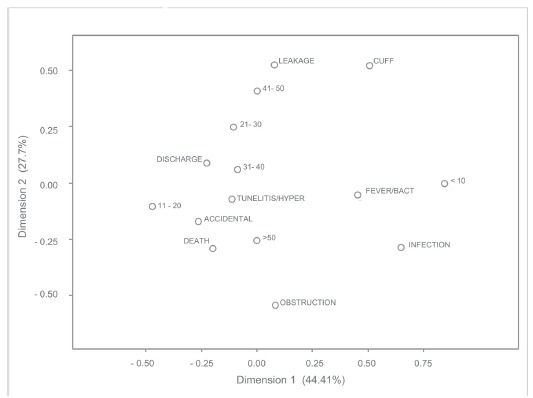
Correspondence between complications of CVC and patient age. São José do Rio Preto, SP, Brazil, 2007-2011

People aged 41-50 years presented the highest incidence of leakage and exteriorization of catheter fixation cuff and the highest number of deaths occurred in those over 50, as shown in Figure 2.

**Figure 3:**
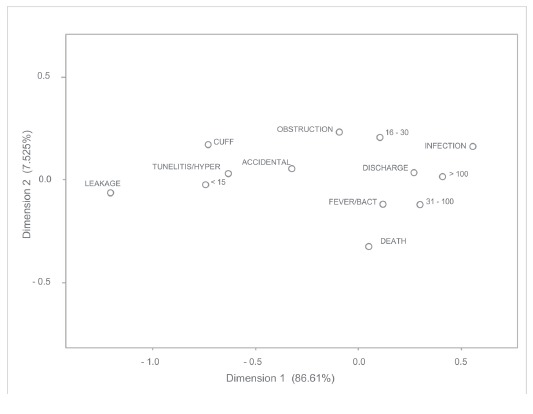
Correspondence between complications of CVC and length of stay with the device. São José do Rio Preto, SP, Brazil, 2007-2011

Regarding complications related to the length of stay with the catheter, tunelitis/hyperemia, exteriorization of catheter fixation cuff and accidental removal were more prevalent in those who remained with the catheter for less than 15 days. From 16 to 30 days, obstruction and between 31 and 100 days, fever/bacteremia, and those patients who were discharged from hospital remained with the catheter for more than 31 days, as shown in Figure 3.

For most patients, hospital discharge occurred with the catheter inserted in the subclavian vein (SCV), regardless of presenting catheter obstruction, accidental removal, tunelitis/hyperemia, infection, and fever/bacteremia. The insertion in the Jugular Vein (JGL) was associated with leakage and in the Femoral Vein (FEM), with the absence of complications.

## Discussion

Hickman catheter was the most commonly used, representing an advance in the management of cancer patients, especially those who need HSCT. Implantation of a long stay CVC of this model is critical to the success of the procedure, since it allows the safe administration of chemotherapy, infusion of Hematopoietic Progenitor Cells (HPC) without compromising the graft, administration of medications, parenteral nutrition, blood products and the safe and comfortable collection of blood samples for tests, reducing costs and complication rates^6^ ^,^ ^8^ ^-^ ^10^.

Complications related to CVC appear immediately, or late, associated with the introduction, permanence and its use. Infection, thrombotic obstruction, exteriorization of catheter fixation cuff, accidental removal, peri-insertion tunelitis/hyperemia, leakage and arrhythmia were identified in 47% of patients with inserted catheters, which corroborates recent studies that show a range of variation from 8 to 69% in the rates of complications related to catheter^11^ ^-^ ^13^.

Infection and obstruction are the most common occurrences and can be caused by multiple factors such as the type of cancer, chemotherapy protocol, caliber of the catheter, insertion site, surgical technique, prior mediastinal irradiation and improper handling by the staff. The age of the patient also exhibited high association with this complication, because the younger the patient, the greater the incidence of infections^6^ ^,^ ^14^ ^-^ ^16^.

Some bacterial infections that occur after autologous transplant are associated with the presence of CVC, although there was indication for its removal as soon as possible. In this group of patients, there is a need to keep it longer for the administration of blood, additional medications, nutrition, intravenous fluids or electrolytes and supplements. After allogeneic transplant, there is a similar risk of infections associated with the use of catheters, mainly bacteremia caused by enteric organisms, resulting from the Graft Versus Host Disease (GVHD) of the intestinal tract and caused by Candida, due to intravenous nutritional supplementation^1^.

Children (under the age of 10 years) showed association with fever/bacteremia and infection, reported in 5.22% of implanted catheters, representing 10% of the identified complications. To set infection related to catheter, it was considered the variation of this data, since 13% of infection were treated as fever/bacteremia resulting from unknown causes.

The incidence of catheter-related infections ranges from 9 to 80%, depending on the model and patient risk factors, as well as its setting. There are those who consider, for a definitive diagnosis, the need to observe the growth of the same microorganism at least in a sample from peripheral blood and from the catheter tip. However there are those who also evaluate the clinical symptomatology^13^ ^,^ ^14^ ^-^ ^18^.

Long-term CVC is also more prone to poor positioning of the distal tip, kinking and thrombosis, with an incidence of 8-20% in patients undergoing autologous stem cell transplantation and lower incidence in the allogeneic and syngeneic^19^. Cancer patients are at increased risk of thromboembolism and often develop blood clots in the catheter, which besides causing obstruction, favors the incidence of infections and represents 3 to 38.3% of complications in adult patients^6^ ^,^ ^14^ ^,^ ^18^.

Regarding the insertion site, there were no significant differences associating complications with the implantation site. Many healthcare centers use CVC in the internal jugular, subclavian and femoral veins, all of which are subject to embolism and bleeding. However, the jugular and subclavian veins have advantages in terms of bacterial contamination, but their disadvantage is the risk of pneumothorax or hemothorax and air embolism due to improper catheter manipulation. The femoral vein is often discouraged due to the high incidence of bacterial infection and thrombosis, in addition to the need of the patient to remain in bed during the time in which the use of the catheter is required^20^ ^-^ ^21^.

In a study of 100 children after HSCT, it was observed that 80 had their catheters successfully implanted in the right or left external jugular, with dissection, which made the technique simple and safe. In this case, the preferred site for CVC implantation was the subclavian vein (76%), which associated to Hickman, provides more comfort to patients who stay for long periods with catheters, alternating between hospitalizations for chemotherapy and home recovery^22^.

Regarding the length of stay with the catheter, 34% continued with the device for a period from 31 to 100 days, with an average of 47.6 days. The longest length of stay was 279 days with a Hickman catheter, which corroborates a comparative study with implants of the same model, which showed an average length of stay of 41.4 days, ranging from 0 to 118 days of stay. The risk of infection is higher in the first 90 days of implantation^1^ ^,^ ^16^ ^,^ ^22^.

Suspected or confirmed infections are the main reason for CVC removal. This is a significant risk factor for bacterial infection in the recovery period. However, premature removal may result in interruption or delay in the treatment, increase in the patient discomfort, anxiety, high costs and increased hospitalization time^16^ ^,^ ^23^ ^-^ ^24^.

Regarding the type of HSCT, complications showed a greater association with the autologous type, although the conditioning method used does not cause immunoablation as in allogeneic transplants that, together, produce hematopoietic pancytopenia and immunocytopenia, favoring the occurrence of opportunistic infections such as those related to CVC^25^.

The occurrence of CVC complications, in relation to the type of transplant, were related to medical diagnosis, since the chemotherapy agents used in most allogeneic conditioning regimens cause dermatitis, rash and skin fragility because they are dermatologically toxic, which can compromise the healing in the insertion site and favor infection. The type of disease, the conditioning regimen and the prior mediastinal irradiation are considered predisposing factors for CVC complications, particularly infection, which was observed in patients with AML and NHL^13^ ^,^ ^15^ ^,^ ^25^.

By choosing CVC, some factors must be considered, such as purpose, expected treatment duration, type of conditioning, training of the team that will handle the device, age of the patient and education level of the caregiver, since most of the time, infections related to CVC can be minimized and/or avoided^23^ ^-^ ^24^.

## Conclusion

Most patients used Hickman catheter, with an average of 47.6 days of implantation and the main complications were fever/bacteremia, accidental removal of CVC, tunelitis/hyperemia around the insertion site and subcutaneous leakage. Regarding the correspondence between the variables, fever/bacteremia was statistically significant in young patients, mostly males, with non-Hodgkin's lymphoma, submitted to an autologous transplant, who remained with the catheter in the subclavian vein for a long period.

The nursing team plays a fundamental role in CVC maintenance and therefore, they must act to prevent injury or complications. In order to prevent fever/bacteremia and infection, nurses must update the multiprofessional team and participate in decisions on the right time for the implant, start of the conditioning regimen, and consider the importance of not extending the period of stay with the catheter.
